# Association of Music Interventions With Health-Related Quality of Life: A Systematic Review and Meta-analysis

**DOI:** 10.1001/jamanetworkopen.2022.3236

**Published:** 2022-03-22

**Authors:** J. Matt McCrary, Eckart Altenmüller, Clara Kretschmer, Daniel S. Scholz

**Affiliations:** 1 Institute of Music Physiology and Musicians’ Medicine, Hannover University of Music, Drama and Media, Hannover, Germany; 2 Prince of Wales Clinical School, University of New South Wales, Sydney, New South Wales, Australia

## Abstract

**Importance:**

Increasing evidence supports the ability of music to broadly promote well-being and health-related quality of life (HRQOL). However, the magnitude of music’s positive association with HRQOL is still unclear, particularly relative to established interventions, limiting inclusion of music interventions in health policy and care.

**Objective:**

To synthesize results of studies investigating outcomes of music interventions in terms of HRQOL, as assessed by the 36- and 12-Item Health Survey Short Forms (SF-36 and SF-12).

**Data Sources:**

MEDLINE, Embase, Web of Science, PsycINFO, ClinicalTrials.gov, and International Clinical Trials Registry Platform (searched July 30, 2021, with no restrictions).

**Study Selection:**

Inclusion criteria were randomized and single-group studies of music interventions reporting SF-36 data at time points before and after the intervention. Observational studies were excluded. Studies were reviewed independently by 2 authors.

**Data Extraction and Synthesis:**

Data were independently extracted and appraised using GRADE criteria (Grading of Recommendations, Assessment, Development, and Evaluations) by multiple authors. Inverse-variance random-effects meta-analyses quantified changes in SF-36 mental and physical component summary (respectively, MCS and PCS) scores from preintervention to postintervention and vs common control groups.

**Main Outcomes and Measures:**

SF-36 or SF-12 MCS and PCS scores, defined a priori.

**Results:**

Analyses included 779 participants from 26 studies (mean [SD] age, 60 [11] years). Music interventions (music listening, 10 studies; music therapy, 7 studies; singing, 8 studies; gospel music, 1 study) were associated with significant improvements in MCS scores (total mean difference, 2.95 points; 95% CI, 1.39-4.51 points; *P* < .001) and PCS scores (total mean difference, 1.09 points; 95% CI, 0.15-2.03 points; *P* = .02). In subgroup analysis (8 studies), the addition of music to standard treatment for a range of conditions was associated with significant improvements in MCS scores vs standard treatment alone (mean difference, 3.72 points; 95% CI, 0.40-7.05 points; *P* = .03). Effect sizes did not vary between music intervention types or doses; no evidence of small study or publication biases was present in any analysis. Mean difference in MCS scores met SF-36 minimum important difference thresholds (mean difference 3 or greater).

**Conclusions and Relevance:**

In this systematic review and meta-analysis, music interventions were associated with clinically meaningful improvements in HRQOL; however, substantial individual variation in intervention outcomes precluded conclusions regarding optimal music interventions and doses for distinct clinical and public health scenarios.

## Key Points

**Question:**

Are music-making and listening interventions associated with positive changes in health-related quality of life?

**Findings:**

This systematic review and meta-analysis of 26 studies comprising 779 individuals found that music interventions were associated with statistically and clinically significant changes in mental HRQOL, both preintervention to postintervention as well as when music interventions were added to treatment as usual vs treatment as usual control groups.

**Meaning:**

These results suggest that associations between music interventions and clinically significant changes in HRQOL are demonstrable in comprehensive reviews of previous studies.

## Teaser

This systematic review and meta-analysis examines the association of a range of therapeutic music interventions with positive changes in health-related quality of life.

## Introduction

Health-related quality of life (HRQOL) is a broad concept capturing “an individual’s or group’s perceived physical and mental health over time.”^1^ HRQOL is closely related to and frequently used interchangeably with well-being,^1^ with the importance of these broad health concepts reflected in their prominence in United Nations Sustainable Development goals: “To ensure healthy lives and promote well-being for all at all ages.”^2^

Listening to and making music (eg, by singing or playing instruments) is increasingly advocated, including in a recent World Health Organization report, as a means of improving HRQOL as well as various domains of well-being in clinical and healthy populations.^3,4,5,6,7^ However, a lack of clarity regarding the magnitude of music effects on HRQOL, particularly compared with other established health interventions, presents clear challenges to the inclusion of music in health policies and care at local, national, and international levels.^8^ Additionally, optimal music intervention types and doses for specific scenarios are still unclear, precluding the formulation of evidence-based music prescriptions.^3,8^

The 36-item Health Survey Short Form (SF-36) HRQOL questionnaire is the most widely used patient-reported outcome instrument in health research, demonstrating strong validity, sensitivity, and reliability across a range of languages, versions (eg, RAND,^9^ Medical Outcomes Study^10^), interventions, and clinical and healthy populations.^9,10,11,12^ Additionally, summary scores from the SF-36 and the reduced 12-item Health Survey Short Form (SF-12) have demonstrated good consistency.^13,14,15^ The SF-36 has also been frequently used in studies of music interventions,^3,4^ providing a means of both quantifying and easily contextualizing the magnitude of music’s association with HRQOL using a broadly valid and applicable instrument. Accordingly, the aim of this study is to quantitatively synthesize and contextualize the associations of music interventions and changes in HRQOL assessed by the SF-36 and SF-12. A secondary study aim is to evaluate these associations relative to specific music intervention types and doses.

## Methods

The protocol for this systematic review and meta-analysis was prospectively registered with PROSPERO (CRD42021276204) and written following the Preferred Reporting Items for Systematic Reviews and Meta-analyses (PRISMA) reporting guideline. This study was exempted from ethics review by the central ethics committee of Leibniz University Hannover because it was a secondary synthesis of deidentified data.

### Search Strategy

Four databases—MEDLINE, EMBASE, Web of Science, and PsycINFO—and 3 clinical trials registries—Cochrane Central Register of Controlled Trials (CENTRAL), ClinicalTrials.gov, and International Clinical Trials Registry Platform (ICTRP)—were searched for peer-reviewed articles, clinical trial registrations, and gray literature reports on July 30, 2021, using the following query: (Music* OR singing OR listening) AND (SF12 OR SF36 OR SF-12 OR SF-36 OR “short form 36” OR “short form 12”). All related subject headings were included where possible and no limitations on study date or language were imposed. The reference lists of included studies and relevant systematic reviews were also hand searched for additional relevant studies.

### Article Screening and Inclusion and Exclusion Criteria

Following removal of duplicate records, titles and abstracts of database search results were screened, followed by full-text review of potentially relevant abstracts against inclusion and exclusion criteria. Screening and full-text review were performed independently in duplicate by 2 study authors (J.M.M. and C.K.), with disagreements resolved through discussion.

Inclusion criteria were randomized and nonrandomized studies investigating the association of music-making (eg, instrumental music, singing, active music therapy) and/or music listening (eg, to recorded or live music, receptive music therapy) interventions with HRQOL in adults using the SF-36 or SF-12 (reduced version) questionnaires. No restrictions were made on eligible control groups. Studies that investigated the association of music with HRQOL as either a primary or secondary objective were both eligible for inclusion. Additionally, studies must have reported the SF-36 or SF-12 Mental Component Summary (MCS) score and/or Physical Component Summary (PCS) score, or data enabling the calculation of a MCS and/or PCS score (eg, data from all 8 subscales included in both the SF-36 and SF-12^10,13^), at both preintervention and immediate postintervention time points. Higher MCS and PCS scores indicated better mental and physical HRQOL, respectively. MCS and PCS from the SF-12 and SF-36 have demonstrated good consistency across a range of populations.^15,16^

MCS and PCS scores are both calculated using norm-based scoring methods including all 8 subscales of the SF-36 and SF-12: physical functioning, role physical, bodily pain, general health, vitality, social functioning, role-emotional, and mental health. In calculations of MCS scores, vitality, social functioning, role-emotional, and mental health subscale scores are given the most weight. Conversely, in calculations of PCS scores, physical functioning, role-physical, bodily pain, and general health subscale scores are given the most weight.^17^ Ware, Kosinsky, and Keller^17^ described MCS and PCS score calculations in further detail, including precise scoring procedures and algorithms. Exclusion criteria were observational and cross-sectional studies and studies that investigated other music-related activities that do not focus on music-making or listening (eg, songwriting).

### Study and Evidence Appraisal (GRADE)

The quality of evidence supporting review conclusions was appraised using the GRADE system. GRADE (Grading of Recommendations, Assessment, Development, and Evaluations) provides a framework for evaluating the risk of bias of individual studies, as well as the level of certainty supporting specific review results.^18^ GRADE was selected for this review because of its broad applicability to different study types and because it has been “designed for reviews…that examine alternative management strategies.”^18^

The risk of bias of individual studies was evaluated using the following standard criteria: allocation concealment, masking (of assessors and data analysts), percentage lost to follow-up, intention-to-treat analysis, selective outcome reporting, use of individual randomization, and control for carryover effects (crossover study design).^19^ Based on these criteria, an evidence quality rating of high, moderate, low, or very low was assigned to each study using established procedures.^19^ All studies were appraised independently by 2 study authors (J.M.M. and C.K.), with any disagreements resolved through discussion. The overall quality and certainty of evidence supporting the results of each meta-analysis was then appraised by the primary author in consultation with the authorship team using the same rating scale.^19^

### Data Extraction

Demographic, music and control intervention, and SF-36 or SF-12 data before and after the intervention were extracted in duplicate by 2 study authors (J.M.M. and C.K.). Data from all available SF-36 and SF-12 subscales were extracted, as well as MCS and PCS summary scores. To maximize consistency of data across studies, MCS and PCS scores were recalculated where possible from underlying subscale data using the methodology of Ware, Kosinsky, and Keller.^17^ Missing MCS and PCS standard deviations were imputed from mental health and physical function subscale scores, respectively, or as medians with minimum and maximum or interquartile range data as per established methods.^20,21^ Authors of studies meeting inclusion criteria but reporting unclear or incomplete SF-36 or SF-12 data were contacted to retrieve compatible MCS and/or PCS data.

### Statistical Analysis

Weighted inverse-variance random-effects meta-analyses were conducted to determine the aggregate pre- to postintervention change in MCS and PCS scores. Additionally, inverse-variance random-effects meta-analyses were performed on postintervention MCS and PCS scores in music vs control groups common to at least 3 studies. The presence of statistical heterogeneity, indicating significant variation in the overall effects of music interventions on MCS and PCS scores, was evaluated using the χ^2^ test and *I^2^* statistic. Potential small study or publication biases were evaluated using the Egger test.^22^ Sensitivity analyses were performed where possible according to music intervention types (eg, music therapy, singing, music listening) and quality of study evidence (very low and low vs moderate and high). Additionally, exploratory nonparametric Spearman correlation analyses were performed to evaluate potential links between key characteristics of the music intervention “dose” (ie, intervention duration, music session frequency and length) and MCS and PCS scores. Significance was set at α = .05 for all statistical tests except meta-analysis main effects; α = .033 was used for meta-analysis main effects to control for multiplicity of related MCS and PCS outcomes, as per recommendations for meta-analyses aiming to best balance Type I and II error risk.^23^ Analyses were conducted in RevMan version 5.4 (Cochrane Collaboration) and SPSS version 26 (IBM Corp).

Finally, published meta-analyses of MCS and PCS scores from established non–pharmaceutical or medical health interventions were retrieved to serve as a basis for comparison with results of the present study. Additionally, changes in MCS and PCS scores were evaluated against a 3-point minimum clinically important difference threshold established by the SF-36 developers.^24^ This threshold was designed to be a general benchmark for meaningful change based on a range of longitudinal and cross-sectional data sets from both clinical and healthy populations; accordingly, this threshold was deemed particularly appropriate for the broad scope and heterogeneous literature included in this review.^24^

## Results

Data from 26 eligible studies and 779 total participants (mean [SD] age, 60 [11] years) were included in the present study (eFigure 1, eTables 1 and 2 in the Supplement). Included studies were conducted in Australia,^25^ Brazil,^26,27,28^ China (Hong Kong SAR),^29^ Germany,^30^ India,^31^ Italy,^32,33,34^ The Netherlands,^35,36^ Spain,^37^ Sweden,^38^ Thailand,^39^ Turkey,^40^ the United Kingdom,^41,42,43,44,45^ and the US.^46,47,48,49,50^ Included studies comprised 22 investigations of clinical populations and 4 of healthy populations (10 investigations examined music listening, 7 music therapy, 8 singing, and 1 gospel music intervention) and 20 randomized clinical trials (RCTs) and 6 single-group studies (8 RCTs included comparisons with a usual treatment control group, 3 RCTs used meditation control groups, and 9 RCTs used a range of other disparate control groups). MCS and PCS scores were available for 25 studies; only MCS data was available in 1 additional study.^49^ Evidence quality was high in 5 studies (19%), moderate in 11 studies (42%), low in 7 studies (27%), and very low in 3 studies (12%) (eTable 3 in the Supplement).

### Pre-Post Changes

**Figure 1.**
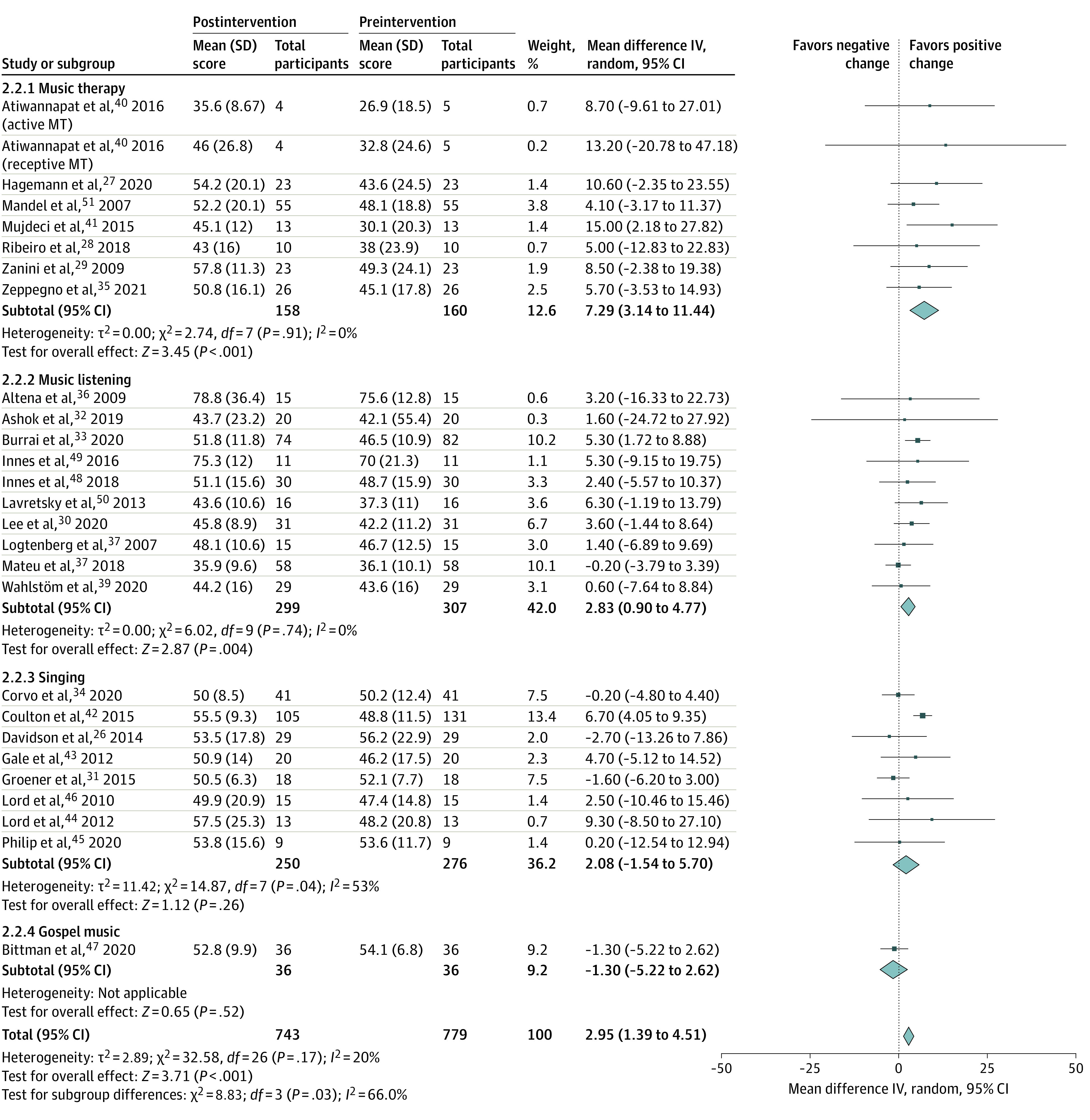
Associations Between Music Interventions and Preintervention to Postintervention Changes in 36-Item and 12-Item Health Survey Short Form Mental Component Summary Scores, Stratified by Music Intervention Type IV indicates inverse variance; MT, music therapy. Box size corresponds to the weighting of each study in the meta-analysis. Diamonds provided for each subgroup as well as the overall analysis indicate the aggregated mean (middle of the diamond) and 95% CIs (points of the diamonds) of results from appropriate included studies. Total refers to the total number of participants included in analyses at preintervention and postintervention time points.

**Figure 2.**
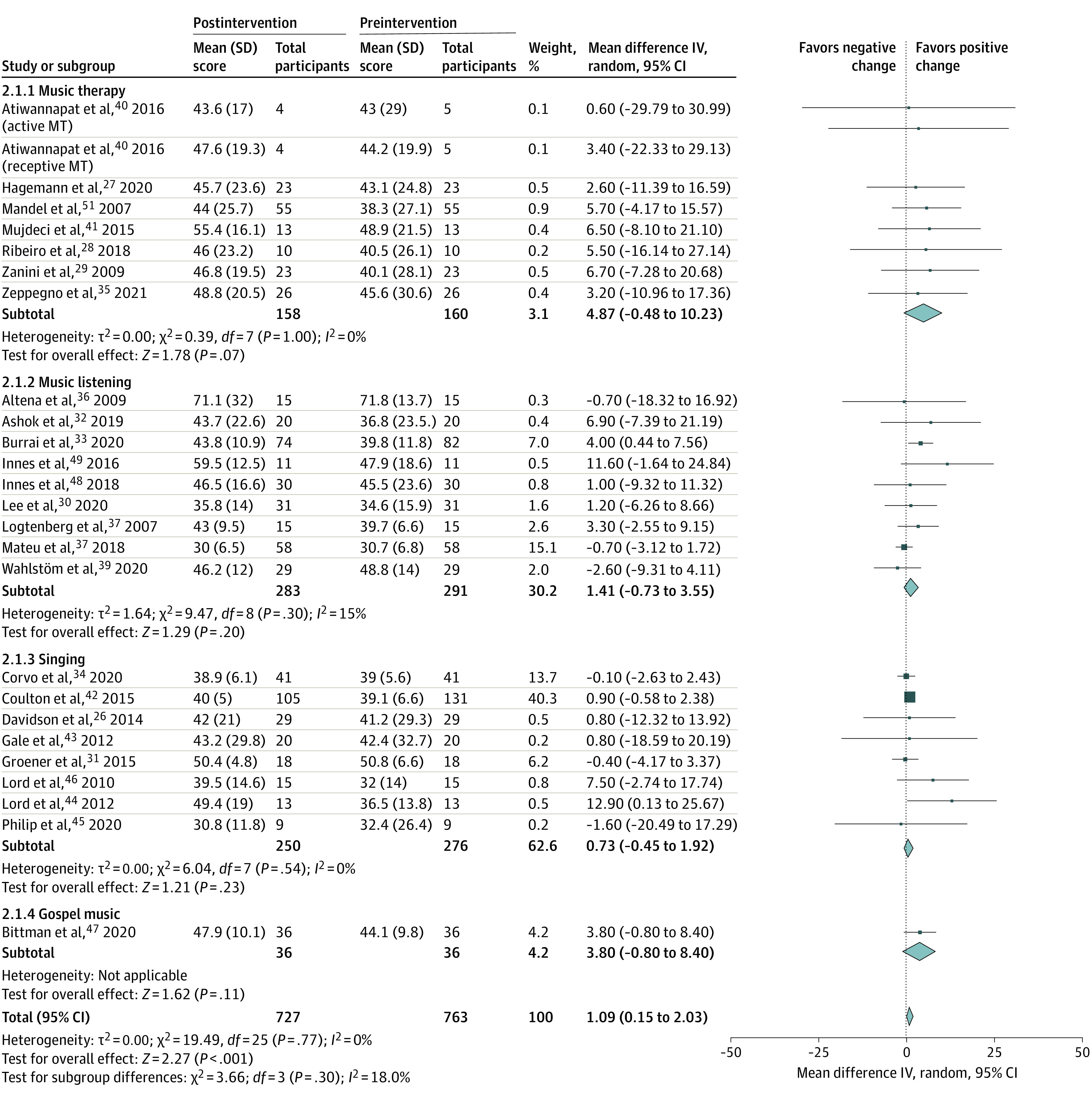
Associations Between Music Interventions and Preintervention to Postintervention Changes in 36-Item and 12-Item Health Survey Short Form Physical Component Summary Scores, Stratified by Music Intervention Type IV indicates inverse variance; MT, music therapy. Total refers to the total number of participants included in analyses at preintervention and postintervention time points.

Music interventions were associated with significant increases in both MCS (total mean difference, 2.95 points; 95% CI, 1.39-4.51 points; *P* < .001) and PCS scores (total mean difference, 1.09 points; 95% CI, 0.15-2.03 points; *P* =.02) from preintervention values (Figure 1 and Figure 2). Standardized mean differences were 0.25 (95% CI, 0.15-0.36) for MCS scores and 0.15 (95% CI, 0.05-0.26) for PCS scores. MCS scores (including 779 participants) were significantly greater in moderate-quality and high-quality vs very low–quality and low-quality studies and varied significantly across intervention types (χ^2^ = 4.56; *I^2^* = 78.1%; *P* = .03) (Figure 1). However, changes in MCS scores did not significantly vary across intervention types when the 1 gospel music intervention study^46^ was excluded. PCS scores (including 763 participants) did not significantly vary according to study quality or intervention type.

No key characteristics of the music intervention dose (ie, intervention duration, music session frequency and length), nor any combination of these characteristics, were associated with changes in MCS or PCS scores. No significant statistical heterogeneity or evidence of small study or publication bias (eFigures 2 and 3 in the Supplement) was present in either analysis. Results of these meta-analyses were also judged to be minimally affected by individual study biases but limited by the imprecision of relatively wide confidence intervals. Accordingly, results were appraised to provide moderate-quality evidence, indicating that “the true effect is probably close to the estimated effect.”^18^

### Music Plus Treatment as Usual vs Treatment as Usual Alone

**Figure 3.**
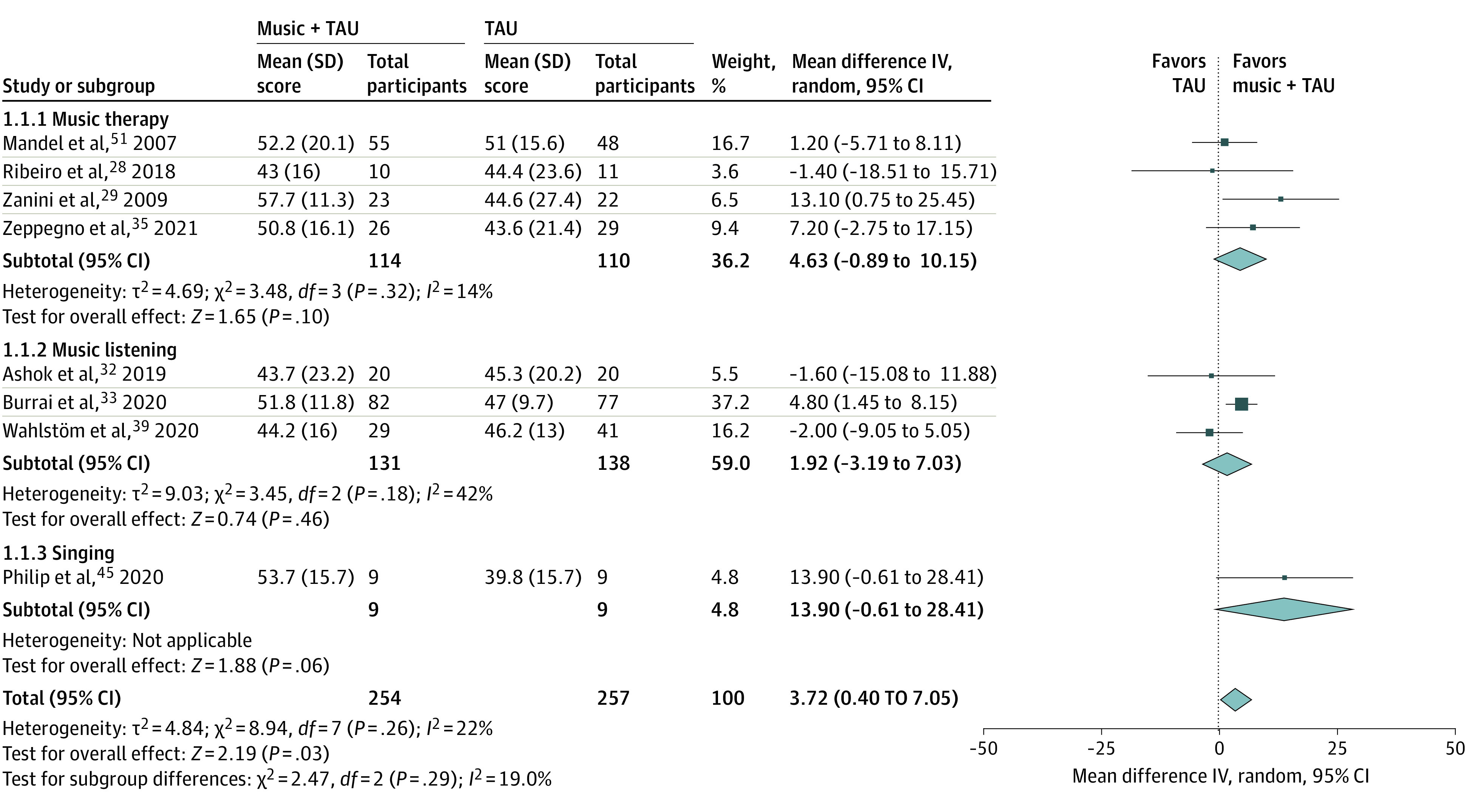
Associations Between Music Interventions Added to Treatment as Usual (TAU) vs TAU Alone and Changes in 36-Item and 12-Item Health Survey Short Form Mental Component Summary Scores, Stratified by Music Intervention Type IV indicates inverse variance. Total refers to the total number of participants included in analyses at preintervention and postintervention time points.

**Figure 4.**
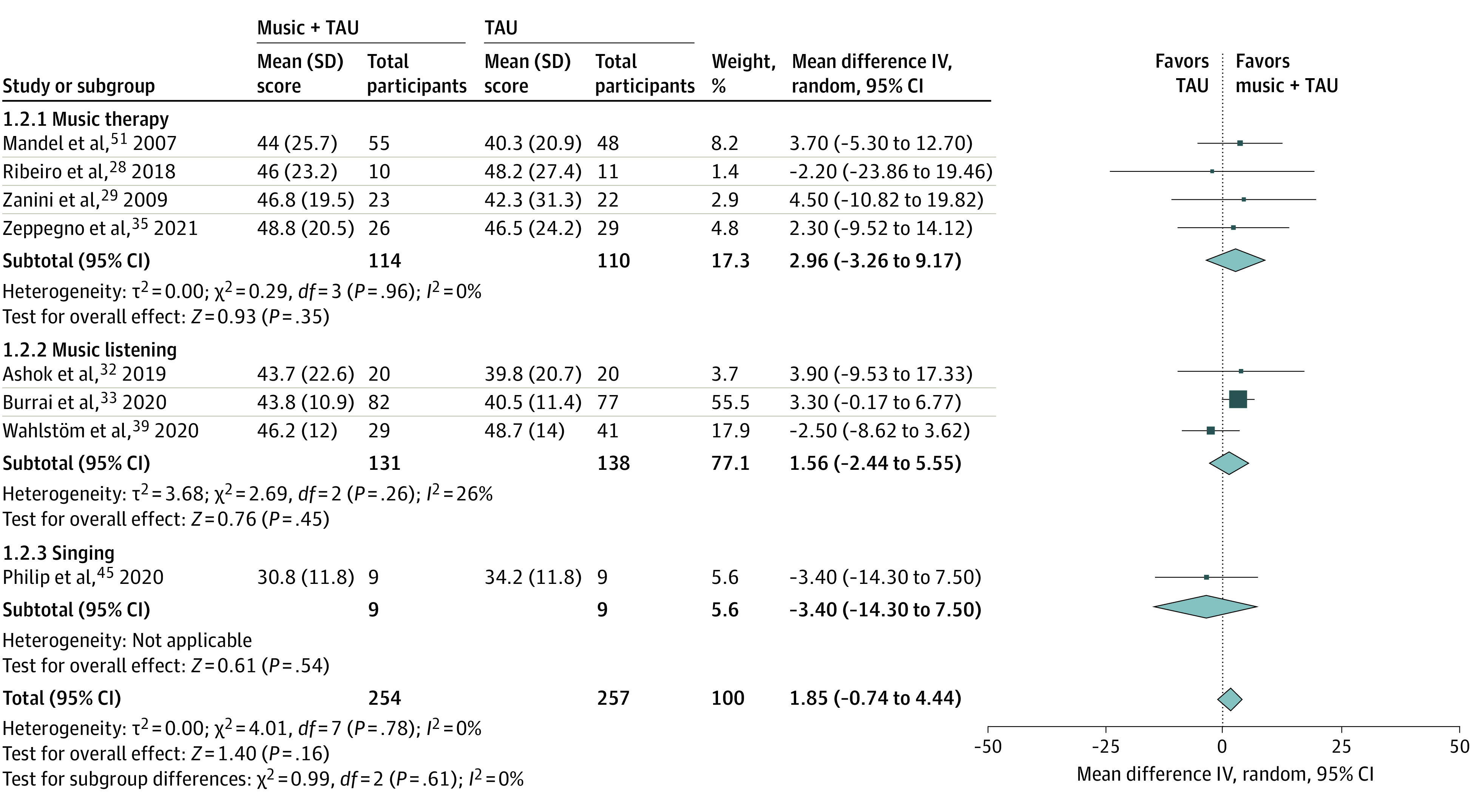
Associations Between Music Interventions Added to Treatment as Usual (TAU) vs TAU Alone and Changes in 36-Item and 12-Item Health Survey Short Form Physical Component Summary Scores, Stratified by Music Intervention Type IV indicates inverse variance. Total refers to the total number of participants included in analyses at preintervention and postintervention time points.

Adding music interventions to treatment as usual (TAU) was associated with significant increases in MCS scores vs TAU alone (total mean difference, 3.72 points; 95% CI, 0.40-7.05 points) (standardized mean difference, 0.24; 95% CI, 0.02-0.45) (*P* = .03) (Figure 3). Differences for PCS scores were not significant (Figure 4). Improved MCS in music plus TAU vs TAU groups did not vary significantly with study quality or music intervention type, and no significant statistical heterogeneity or evidence of small study or publication biases was present in either analysis (eFigures 4 and 5 in the Supplement). Pre-post intervention changes in MCS and PCS scores associated with music plus TAU interventions did not significantly differ from changes in MCS and PCS scores associated with all other included music interventions (eFigures 6 and 7 in the Supplement). Results of these meta-analyses were judged to be minimally affected by individual study biases but limited by the imprecision of wide confidence intervals across studies. Accordingly, results were appraised to provide moderate-quality evidence.

### Music Listening vs Meditation

No significant differences in MCS or PCS scores in music listening vs meditation intervention studies were present across 3 included studies (eFigures 8 and 9 in the Supplement). Once again, no significant statistical heterogeneity or evidence of small study or publication biases was present in either analysis. However, results were limited by the small number of studies and wide confidence intervals, and judged to provide low-quality evidence (ie, “the true effect might be markedly different from the estimated effect”).^18^

### HRQOL Changes Associated With Music Interventions in Context

Changes in MCS scores, both pre-post intervention and vs TAU, met or exceeded the proposed 3-point minimum important difference threshold for MCS and PCS scores.^17^ Pre-post changes in PCS scores (1.1-point improvement) fell below this threshold.

Changes in MCS scores (pre-post and vs TAU) were similar to changes in PCS scores reported for weight loss in studies of adults with obesity (2.8-point improvement; no significant MCS change).^51^ However, mean differences in MCS and PCS scores (pre-post and vs TAU) associated with music interventions were substantially smaller than differences in MCS and PCS scores associated with resistance exercise (ie, strength training) in older adults from mixed clinical and healthy populations vs mixed control groups (standardized mean difference: MCS, 0.54; PCS, 0.50)^52^ and mixed modes of exercise in participants with knee osteoarthritis vs inactive or psycho-educational control groups (standardized mean difference: MCS, 0.44; PCS, 0.52).^53^

## Discussion

This meta-analysis of 26 studies of music interventions provided clear and quantitative moderate-quality evidence that music interventions are associated with clinically significant changes in mental HRQOL. Additionally, a subset of 8 studies demonstrated that adding music interventions to usual treatment was associated with clinically significant changes to mental HRQOL in a range of conditions. Music interventions were associated with substantially smaller changes in physical HRQOL, which are of potentially equivocal practical importance.^17^ The substantial individual variation in responses to music interventions across included studies should be emphasized; this analysis must only be used as a general guide to the associations between music interventions and HRQOL changes.

Included studies presented considerable heterogeneity in study populations and geographic locations, music intervention types and doses, and TAU control groups. However, no statistical heterogeneity or evidence of small study or publication bias was present in any analyses. This suggests that results approximate the true, albeit general, association between music interventions and changes in HRQOL. Further research is still needed to provide guidance regarding optimal music interventions and doses in distinct clinical and public health scenarios.

Associations between music interventions and changes in MCS scores (pre-post and music plus TAU vs TAU) are within the range, albeit on the low end, of changes in MCS and PCS scores associated with established non–pharmaceutical/medical,^51,52,53,54^ as well as pharmaceutical/medical,^55,56,57^ health interventions, and thus are likely to be clinically significant.^17^ Accordingly, this review quantitatively confirmed narrative syntheses from prior systematic reviews asserting that music interventions are linked to meaningful improvements in well-being and HRQOL.^3,4,5,6^ Of particular interest for future study and health policy is the fact that these benefits are associated with participation in a broadly rewarding activity.^58^ While uptake and adherence challenges persist with other non–pharmaceutical/medical interventions (eg, weight loss, exercise),^59,60^ music is “reliably ranked as one of life’s greatest pleasures.”^61^ As such, music interventions may present a more attractive and effective nonpharmaceutical alternative to other health interventions. Further study is required to investigate this hypothesis and clarify the specific utility of music vs other established interventions.

Additionally, targeted research is also needed to provide insights into the mechanisms of music interventions’ association with positive changes in HRQOL—ie, the who, what, when, where, and how underpinning their effectiveness. The absence of any significant differences between music intervention types and doses in the present analyses is intriguing but not definitive; these results could also be simply explained by the diversity of included populations and interventions, even within specific intervention types (particularly clearly demonstrated for music listening interventions in the Table). Broad confidence intervals of both main and intervention type–specific results in this meta-analysis likely also reflect the diversity of interventions. A 2021 analysis^62^ indicated that the mechanisms of music’s impact on health are complex and specific to distinct settings, suggesting that targeted study is required to determine optimal music intervention characteristics in each setting. However, other analyses propose that such targeted research may be able to be rapidly generalized to other settings if foundational physiological mechanisms of music intervention effects can be identified and targeted.^63^

**Table.** Characteristics of Included Studies

Source	Study design	Population	Music intervention				Music intervention group			Control group	
			Type	Length	Session frequency	Session duration	Total, No.	Men/women	Mean age, y	Type	Total, No.
Altena et al,^35^ 2009	RCT	Clinical (hypertension)	Music listening (“slow music”)	9 wk	Daily	Not specified	15	8/7	59	Exercises with breathing device	15
Ashok, Shanmugam, and Soman,^31^ 2019	RCT	Clinical (coronary bypass)	Music listening (sedative music without lyrics with tempo 60-80 beats per minute) + TAU	1 wk	Daily	20 min	20	6/14	60.8	TAU (cardiac rehabilitation)	20
Atiwannapat et al,^39^ 2016	RCT	Clinical (major depressive disorder)	Music therapy (individual, active)	12 wk	Weekly	1 h	5	1/4	41.6	Group counselling	4
			Music therapy (individual, receptive)	12 wk	Weekly	1 h	5	2/3	54.4	Group counselling	4
Bittman et al,^46^ 2020	RCT	Clinical (2 or more metabolic risk factors)	Gospel music program (singing and playing musical instruments [clavinovas, guitars, drums]) + health education	1 y	Weekly	45 min	36	6/30	62.5	Health education (cardiovascular risk reduction)	35
Burrai et al,^32^ 2020	RCT	Clinical (heart failure)	Music listening (recorded classical music; experimenter-selected tracks with tempo 60-80 beats per minute)	12 wk	Daily	30 min	82	47/35	71.6	TAU (heart failure)	77
Corvo, Skingley, and Clift,^33^ 2020	Single group study	Healthy (older people)	Singing (1 singing group	12 wk	Weekly	2 h	41	“Predominantly female”	No data	NA	NA
Coulton et al,^41^ 2015	RCT	Healthy (older people)	Singing (group)	14 wk	Weekly	1.5 h	131	25/106	69.2	Wait list	127
Davidson et al,^25^ 2014	Single group study	Healthy (older people)	Singing (group)	8 wk	Weekly	1.5 h	29	21/8	77.5	NA	NA
Gale et al,^42^ 2012	Single group study	Clinical (cancer survivors)	Singing (group)	12 wk	Weekly	2 h	30	Unspecified	60.2	NA	NA
Groener et al,^30^ 2015	RCT	Clinical (diabetes)	Singing (group) + Health education	3 d	Daily	30 min	18	14/4	46	Health education	17
Hagemann, Martin, and Neme,^26^ 2020	Single group study	Clinical (chronic kidney disease)	Music therapy (group, active)	4 wk	Twice weekly	1.25 h	23	12/11	54.9	NA	NA
Innes et al,^47^ 2018	RCT	Clinical (knee osteoarthritis)	Music listening (recorded classical music; patient choice of 80 experimenter-selected songs)	8 wk	Twice daily	20 min	11	5/6	58.8	Meditation (Mantra)	11
Innes et al,^48^ 2016	RCT	Clinical (cognitive decline)	Music listening (recorded classical music; patient choice of 80 experimenter-selected songs)	12 wk	Daily	12 min	30	5/25	60.2	Meditation (Kirtan Kriya)	30
Lavretsky et al,^49^ 2013	RCT	Clinical (dementia caregivers with depressive symptoms)	Music listening (recorded music; experimenter provided CD)	8 wk	Daily	12 min	16	2/14	60.6	Meditation (Kirtan Kriya)	23
Lee, Chan, Mok^29^ 2010	RCT	Healthy (older people)	Music listening (Patient choice of experimenter-selected music; meditative, Asian classical, Western classical, slow jazz, Chinese classical)	4 wk	Weekly	30 min	31	11/20	75.5	Inactive	35
Logtenberg et al,^36^ 2007	RCT	Clinical (type 2 diabetes + hypertension)	Music listening (“various kinds of random music”)	8 wk	Daily	Not specified	15	3/12	62.7	Exercises with breathing device	15
Lord et al,^45^ 2010	RCT	Clinical (COPD)	Singing (group) + breathing education	6 wk	Twice weekly	1 h	15	No data	66.6	Breathing education alone	13
Lord et al,^43^ 2012	RCT	Clinical (COPD)	Singing (group) + breathing education	8 wk	Twice weekly	1 h	13	No data	68.6	Film workshops + breathing education	11
Mandel et al,^50^ 2007	RCT	Clinical (cardiac rehabilitation)	Music therapy (active, individual) + TAU	10 wk	Every other week	1.5 h	55	27/28	65	TAU (cardiac rehabilitation)	48
Mateu et al,^37^ 2018	Single group crossover study	Clinical (low-back pain)	Music listening (relaxing music; patient choice of songs from provided CD)	8 wk	Daily	Not specified	58	15/43	51	Progressive muscle relaxation with ‘low-level background music’	58
Mujdeci et al,^40^ 2015	Single group study	Clinical (tinnitus)	Music therapy (receptive; listening to patient preferred recorded music, edited to be 70% music and 30% noise)	8 wk	Daily	2 h	13	7/6	46.8	NA	NA
Philip et al,^44^ 2020	RCT	Clinical (COPD)	Singing (group) + TAU	12 wk	Weekly	1 h	9	6/3	72.1	TAU (COPD)	9
Ribeiro,^27^ 2018	RCT	Clinical (NICU mothers)	Music therapy (individual; receptive) + TAU	7 wk	Weekly	45 min	10	0/10	25.8	TAU (NICU)	11
Wahlstöm et al,^38^ 2020	RCT	Clinical (atrial fibrillation)	Music listening (relaxing recorded music (MediCure) delivered in group setting) + TAU	12 wk	Weekly	30 min	29	14/15	64	TAU (atrial fibrillation)	41
Zanini et al,^28^ 2009	RCT	Clinical (hypertension)	Music therapy (group, active) + TAU	12 wk	Weekly	1 h	23	7/16	66.5	TAU (hypertension)	22
Zeppegno et al,^34^ 2021	RCT	Clinical (breast cancer)	Music therapy (group, active) + TAU	6 wk	Weekly	1 h	26	No data	No data	TAU (radiotherapy)	29

Abbreviations: COPD, chronic obstructive pulmonary disorder; NA, not applicable; NICU, neonatal intensive care unit; RCT, randomized controlled trial; TAU, treatment as usual.

### Limitations

This study had several limitations. Review was limited by its broad inclusion criteria that limited conclusions regarding the associations of specific music interventions in particular scenarios with specific HRQOL changes, especially given the diversity of included interventions. Despite this limitation, which would preclude the conduct of many meta-analyses, we contend that our meta-analysis was justified by the demonstrated need for even general quantitative syntheses, which allow music effects to be clearly contextualized.^8^ Additionally, standardized mean differences describing the magnitude of pre-post intervention effects have been shown to be prone to bias and must be interpreted with caution.^64^ However, the similar effect sizes of changes in MCS scores in pre-post and music plus TAU vs TAU analyses provided additional confidence in the average magnitude of pre-post MCS changes. Finally, this review was ultimately limited to studies evaluating the association of music interventions HRQOL using the SF-36 or SF-12 instruments, a possibly skewed subset of music intervention studies. Statistical homogeneity, the absence of apparent publication or small study biases, and the broad psychometric rigor of the SF-36 and SF-12^15,16^ suggest that results of this review approximated the true associations between music interventions and HRQOL changes. However, the possibility remains that this subset of studies was not representative of music’s general effects on HRQOL or that the SF-36 and SF-12 instruments do not completely capture the impact of music on HRQOL. This uncertainty is reflected in the moderate quality rating of key review results, indicating that “the true effect is probably close to the estimated effect.”^18^

## Conclusions

This study provided moderate-quality quantitative evidence of associations between music interventions and clinically significant changes in mental HRQOL. Mean differences in physical HRQOL associated with music interventions were potentially equivocal. Changes in mental HRQOL associated with music interventions were within the range, albeit at the low end, of average effects of established non–pharmaceutical and medical interventions (eg, exercise, weight loss). Substantial individual variation in music intervention effects precluded conclusions regarding music use in specific scenarios. Future research is needed to clarify optimal music interventions and doses for use in specific clinical and public health scenarios.

## Notes

Supplement. **eTable 1.** Articles Excluded After Full-text Review, With Reasons **eTable 2.** Reviews Searched for Additional Records in This Meta-analysis **eTable 3.** GRADE Quality of Evidence Ratings for Included Studies **eFigure 1.** PRISMA Flow Diagram **eFigure 2.** Funnel Plot Detailing Distribution of MCS Pre-post Changes **eFigure 3.** Funnel Plot Detailing Distribution of PCS Pre-post Changes **eFigure 4.** Funnel Plot Detailing Distribution of MCS Changes in Music Plus TAU vs TAU Interventions **eFigure 5.** Funnel Plot Detailing Distribution of PCS Changes in Music Plus TAU vs TAU Interventions **eFigure 6.** Meta-analysis of Pre-post Changes in MCS Scores, Stratified by Music Plus TAU vs All Other Interventions **eFigure 7.** Meta-analysis of Pre-post Changes in PCS Scores, Stratified by Music Plus TAU vs All Other Interventions **eFigure 8.** Meta-analysis of MCS Scores in Music vs Meditation Interventions **eFigure 9.** Meta-analysis of PCS Scores in Music vs Meditation Interventions
