# Supplementary material for: Association of Music Interventions With Health-Related Quality of Life: A Systematic Review and Meta-analysis
Source: JAMA Netw Open. 2022 Mar 22;5(3):e223236. doi: 10.1001/jamanetworkopen.2022.3236 (PMC8941357; doi:10.1001/jamanetworkopen.2022.3236)
Supplement: Supplement. — eTable 1. Articles Excluded After Full-text Review, With Reasons eTable 2. Reviews Searched for Additional Records in This Meta-analysis eTable 3. GRADE Quality of Evidence Ratings for Included Studies eFigure 1. PRISMA Flow Diagram eFigure 2. Funnel Plot Detailing Distribution of MCS Pre-post Changes eFigure 3. Funnel Plot Detailing Distribution of PCS Pre-post Changes eFigure 4. Funnel Plot Detailing Distribution of MCS Changes in Music Plus TAU vs TAU Interventions eFigure 5. Funnel Plot Detailing Distribution of PCS Changes in Music Plus TAU vs TAU Interventions eFigure 6. Meta-analysis of Pre-post Changes in MCS Scores, Stratified by Music Plus TAU vs All Other Interventions eFigure 7. Meta-analysis of Pre-post Changes in PCS Scores, Stratified by Music Plus TAU vs All Other Interventions eFigure 8. Meta-analysis of MCS Scores in Music vs Meditation Interventions eFigure 9. Meta-analysis of PCS Scores in Music vs Meditation Interventions [file jamanetwopen-e223236-s001.pdf]

Supplemental Online Content

McCrary JM, Altenmüller E, Kretschmer C, Scholz DS. Association of music interventions with health-related quality of life: a systematic review and meta-analysis. *JAMA Netw Open.* 2022;5(3):e223236. doi:10.1001/jamanetworkopen.2022.3236

eTable 1. Articles Excluded After Full-text Review, With Reasons

eTable 2. Reviews Searched for Additional Records in This Meta-analysis

eTable 3. GRADE Quality of Evidence Ratings for Included Studies

eFigure 1. PRISMA Flow Diagram

eFigure 2. Funnel Plot Detailing Distribution of MCS Pre-post Changes

eFigure 3. Funnel Plot Detailing Distribution of PCS Pre-post Changes

eFigure 4. Funnel Plot Detailing Distribution of MCS Changes in Music Plus TAU vs TAU Interventions

eFigure 5. Funnel Plot Detailing Distribution of PCS Changes in Music Plus TAU vs TAU Interventions

eFigure 6. Meta-analysis of Pre-post Changes in MCS Scores, Stratified by Music Plus TAU vs All Other Interventions

eFigure 7. Meta-analysis of Pre-post Changes in PCS Scores, Stratified by Music Plus TAU vs All Other Interventions

eFigure 8. Meta-analysis of MCS Scores in Music vs Meditation Interventions

eFigure 9. Meta-analysis of PCS Scores in Music vs Meditation Interventions

This supplemental material has been provided by the authors to give readers additional information about their work.

© 2022 McCrary JM et al. *JAMA Network Open.*

eTable 1. Articles excluded after full-text review, with reasons.

Author	Year	Title	Journal	Reason for Exclusion
Akmese & Oran	2014	Journal of Midwifery and Women's Health	Effects of Progressive Muscle Relaxation Exercises Accompanied by Music on Low Back Pain and Quality of Life During Pregnancy	Not a music-focused intervention
Ashok & Soman	2018	International Journal of Pharma and Bio Sciences	Efficacy of music therapy on hospital induced anxiety and health related quality of life in coronary artery bypass graft patients: Study protocol for a randomized controlled trial	Study protocol for Ashok, Shanmugam & Soman 2019 (included)
Bell, McIntyre & Hadley	2016	Psychomusicology: Music, Mind and Brain	Listening to classical music results in a positive correlation between spatial reasoning and mindfulness	No use of SF-36/12
Bidabadi & Mehryar	2015	Journal of Affective Disorders	Music therapy as an adjunct to standard treatment for obsessive compulsive disorder and co-morbid anxiety and depression: A randomized clinical trial	No use of SF-36/12
Bygren et al.	2009	Psychosomatic Medicine	Cultural participation and health: A randomized controlled trial among medical care staff	No compatible SF-36 data; SF-36 data only available as a composite of multiple interventions
Cao et al.	2016	International Journal of Clinical and Experimental Medicine	Music therapy improves pregnancy-induced hypertension treatment efficacy	No pre and post-test SF-36 data
Castelino et al.	2013	Australasian Psychiatry	The effect of group music therapy on anxiety, depression and quality of life in older adults with psychiatric disorders	No pre and post-test SF-36 data collection (data only at pre- and 4-weeks post-intervention completion)
Cohen et al.	2006	The Gerontologist	The Impact of Professionally Conducted Cultural Programs on the Physical Health, Mental Health, and Social Functioning of Older Adults	No use of SF-36/12
Cooke et al.	2010	Aging & Mental Health	A randomized controlled trial exploring the effect of music on agitated behaviours and anxiety in older people with dementia	No use of SF-36/12
Erkkila et al.	2011	British Journal of Psychiatry	Individual music therapy for depression: A randomised controlled trial	No compatible SF-36 data available
Erkkila et al.	2021	Frontiers in Psychology	Music Therapy for Depression Enhanced With Listening Homework and Slow Paced Breathing: A Randomised Controlled Trial	No compatible SF-36 data available
Franco et al.	2014	Psychology of Music	Affect-matching music improves cognitive performance in adults and young children for both positive and negative emotions	No use of SF-36/12
Gold et al.	2013	Psychotherapy & Psychosomatics	Individual music therapy for mental health care clients with low therapy motivation: Multicentre randomised controlled trial	No use of SF-36/12
Hattori et al.	2011	Geriatrics & Gerontology International	Controlled study on the cognitive and psychological effect of coloring and drawing in mild Alzheimer's disease patients	Not a music-focused intervention
Heiderscheit	2006	Thesis	The effects of the Bonny Method of Guided Imagery and Music on interpersonal problems, sense of coherence and salivary immunoglobulin a of adults in chemical dependency treatment	No use of SF-36/12
Henneghan & Becker	2019	Archives of Physical Medicine and Rehabilitation	Improving Cognitive and Psychosocial Symptoms and Social Functioning in Breast Cancer Survivors	No compatible SF-36 data available in conference abstract (social subscale reported only)

© 2022 McCrary JM et al. *JAMA Network Open.*

Hofmann et al.	2010	17th International Congress on Sound & Vibration	Additional effects of multisensory perception of music with a vibroacoustic mat to pure listening of music	No use of SF-36/12 (use of modified SF-12)
Hseih et al.	2019	European Journal of Cancer Care	Effect of home-based music intervention versus ambient music on breast cancer survivors in the community: A feasibility study in Taiwan	No use of SF-36/12
Innes et al.	2018	Journal of Alzheimer's Disease	Effects of meditation and music-listening on blood biomarkers of cellular aging and Alzheimer's disease in adults with subjective cognitive decline: An exploratory randomized clinical trial	Another report of study described in Innes et al. 2016 (included) with fewer participants
Innes et al.	2016	Complementary Therapies in Medicine	A randomized controlled trial of two simple mind-body programs, Kirtan Kriya meditation and music listening, for adults with subjective cognitive decline: Feasibility and acceptability	Another report of study described in Innes et al. 2018 (included)
Jeon, Kim & Yoo	2009	Journal of Korean Academy of Nursing	Effects of music therapy and rhythmic exercise on quality of life, blood pressure and upper extremity muscle strength in institution-dwelling elderly women	No compatible SF-36 data available
Kim & Kang	2021	Geriatric Nursing	Effect of a group music intervention on cognitive function and mental health outcomes among nursing home residents: A randomized controlled pilot study	No use of SF-36/12
Liddle, Parkinson & Sibbritt	2012	Australasian Journal on Ageing	Painting pictures and playing musical instruments: Change in participation and relationship to health in older women	Not an intervention study
Lin et al.	2020	Annals of Thoracic and Cardiovascular Surgery	Effect of music therapy on the chronic pain and midterm quality of life of patients after mechanical valve replacement	No pre and post-test SF-36 data
Lord et al.	2010	American Journal of Respiratory and Critical Care Medicine	Effect of singing lessons in patients with COPD - A randomised controlled trial	Abstract version of Lord et al. 2012 (included)
Lord et al.	2012	American Journal of Respiratory and Critical Care Medicine	Effects of "singing for breathing" TM in patients with chronic obstructive pulmonary disease (COPD)-a randomized control trial	Abstract version of Lord et al. 2012 (included)
Lord et al.	2011	Journal of Aerosol Medicine and Pulmonary Drug Delivery	Singing for breathing effects of singing lessons in patients with COPD - a randomised control trial	Another report of study described in Lord et al. 2012 (included)
Low et al.	2020	Journal of Alternative and Complementary Medicine	Vocal music therapy for chronic pain: A mixed methods feasibility study	No use of SF-36/12
Mandel et al.	2007	Journal of Music Therapy	Effects of music therapy on health-related outcomes in cardiac rehabilitation: A randomized controlled trial	No pre and post-test SF-36 data
Mandel, Davis & Secic	2014	Hospital Topics	Effects of music therapy on patient satisfaction and health-related quality of life of hospital inpatients	No pre and post-test SF-36 data
Mateu et al.	2012	Basic and Clinical Pharmacology and Toxicology	Jacobson's progressive muscle relaxation as adjunctive therapy in osteoarticular chronic pain	Abstract version of Mateu et al. 2018 (included)
Novotna et al.	2017	Multiple Sclerosis	Effect of music therapy on common symptoms of multiple sclerosis	No compatible SF-36 data available in conference abstract
Pearce et al.	2016	Journal of Community & Applied Social Psychology	Is group singing special? Health, well-being and social bonds in community-based adult education classes	No use of SF-36/12 (use of modified SF-36)
Poćwierz-Marciniak & Bidzan	2017	Health Psychology Report	The influence of music therapy on quality of life after a stroke	No compatible SF-36 data available

© 2022 McCrary JM et al. *JAMA Network Open.*

Puhan et al.	2006	BMJ	Didgeridoo playing as alternative treatment for obstructive sleep apnea syndrome: randomised controlled trial	No pre and post-test SF-36 data
Raglio et al.	2016	International Journal of Neuroscience	Improvement of spontaneous language in stroke patients with chronic aphasia treated with music therapy: A randomized controlled trial	No compatible SF-36 data available
Reagon et al.	2017	European Journal of Cancer Care	Choir singing and health status in people affected by cancer	Observational, not intervention study
Ross, Hollen & Fitzgerald	2006	American Journal of Kidney Disease	Observational study of an Arts-in-Medicine Program in an outpatient hemodialysis unit	Not a music-focused intervention
Russ et al.	2020	Journal of Alternative and Complementary Medicine	Cortisol as an acute stress biomarker in young hematopoietic cell transplant patients/caregivers: Active music engagement protocol	No use of SF-36/12
Shiranbidabadi & Mehryar	2015	Journal of Affective Disorders	Music therapy as an adjunct to standard treatment for obsessive compulsive disorder and co-morbid anxiety and depression: A randomized clinical trial	No use of SF-36/12
Skingley et al.	2011	BMC Public Health	The effectiveness and cost-effectiveness of a participative community singing programme as a health promotion initiative for older people: protocol for a randomised controlled trial	Another report of study described in Coulton et al. 2015 (included)
Skingley et al.	2014	Arts & Health	Singing for breathing: Participants' perceptions of a group singing programme for people with COPD	Another report of study described in Coulton et al. 2015 (included)
Skingley, Marin & Clift	2016	Journal of Applied Gerontology	The contribution of community singing groups to the well-being of older people: Participant perspectives from the United Kingdom	Another report of study described in Coulton et al. 2015 (included)
Tai, Wang & Yang	2015	Neuropsychiatric Disease and Treatment	Effect of music intervention on the cognitive and depression status of senior apartment residents in Taiwan	No use of SF-36/12
Unspecified (https://trialsearch.who.int/Trial2.aspx?TrialID=ACTRN12616001671459)	2016	ACTRN Clinical Trials Registry	Personalised relaxation practice to improve sleep quality in patients with chronic fatigue syndrome and depression: a Randomised Control Trial	No study results available
Unspecified (https://trialsearch.who.int/Trial2.aspx?TrialID=ISRCTN50156343)	2012	ISRCTN	Music and expressive arts therapy for women with a history of gynaecological cancer	No use of SF-36/12
Unspecified (http://www.who.int/trialsearch/Trial2.aspx?TrialID=ACTRN12614000168651)	2014	ACTRN Clinical Trials Registry	Music Therapy for Older Adults	Not a music-focused intervention
Unspecified (http://www.who.int/trialsearch/Trial2.aspx?TrialID=DRKS00024549)	2021	DRKS (clinical trials registry)	Effects of Receptive Music Therapy with a Monochord in multiple sclerosis (MUTIMS) – a randomized controlled study	Study ongoing

© 2022 McCrary JM et al. *JAMA Network Open.*

Unspecified (http://www.who.int/trialsearch/Trial2.aspx?TrialID=ISRCTN42943709)	2019	ISRCTN	Singing and COPD: a pilot randomised controlled trial	Study ongoing
Unspecified (https://clinicaltrials.gov/show/NCT00500526)	2007	Clinicaltrials.gov	Effects of Singing in Chronic Obstructive Pulmonary Disease	No use of SF-36, confirmed through author contact
Unspecified (https://clinicaltrials.gov/show/NCT03076801)	2017	Clinicaltrials.gov	Does Choral Singing Help Improve Stress in Patients With Ischemic Heart Disease?	No study results available
Unspecified (https://clinicaltrials.gov/show/NCT04034212)	2019	Clinicaltrials.gov	Singing for Health: improving Experiences of Lung Disease (SHIELD Trial)	Study ongoing
Unspecified (https://clinicaltrials.gov/show/NCT04446624)	2020	Clinicaltrials.gov	Oxidative Stress, Anxiety and Depression in Breast Cancer Patients: impact of Music Therapy	Not a music-focused intervention
Unspecified (https://clinicaltrials.gov/show/NCT04638244)	2020	Clinicaltrials.gov	Brief Online Music Intervention (BOMI) in Improving the Mental Well-being of Young People in the Community in Hong Kong	Study ongoing
Vara et al.	2020	International Urogynecology Journal	Music therapy in rehabilitation treatment for chronic pelvic pain	No compatible SF-36 data available in conference abstract
Wahlstöm et al.	2018	Circulation	Mediyoga improves health related quality of life and blood pressure among patients with paroxysmal atrial fibrillation-the MYPAF study	Abstract version of Wählstrom et al. 2020 (included)
Zanini et al.	2010	Journal of Hypertension	Music therapy contributing to the quality of life of hypertensive patients	Abstract version of Zanini et al. 2009 (included)
Zheng & Zhang	2020	Basic and Clinical Pharmacology and Toxicology	Effect of Music on Novel Coronavirus Pneumonia Patients' Rehabilitation Training after Recovery	No compatible SF-36 data available in conference abstract

© 2022 McCrary JM et al. *JAMA Network Open.*

eTable 2. Review articles included at full-text article review stage. Citations of these reviews were searched for additional relevant articles.

Author	Year	Journal	Title
Archer, Buxton & Sheffield	2015	Psycho-Oncology	The effect of creative psychological interventions on psychological outcomes for adult cancer patients: A systematic review of randomised controlled trials
Bradt & Dileo	2014	Cochrane Database of Systematic Reviews	Music interventions for mechanically ventilated patients
Bradt et al.	2016	Cochrane Database of Systematic Reviews	Music interventions for improving psychological and physical outcomes in cancer patients
Bradt, Dileo & Potvin	2013	Cochrane Database of Systematic Reviews	Music for stress and anxiety reduction in coronary heart disease patients
Campbell, Bodkin-Allen & Swain	2021	Journal of Health Psychology	Group singing improves both physical and psychological wellbeing in people with and without chronic health conditions: A narrative review
Geretsegger et al.	2014	Cochrane Database of Systematic Reviews	Music therapy for people with autism spectrum disorder
Geretsegger et al.	2017	Cochrane Database of Systematic Reviews	Music therapy for people with schizophrenia and schizophrenia-like disorders
Jespersen et al.	2015	Cochrane Database of Systematic Reviews	Music for insomnia in adults
McNamaral et al.	2017	Cochrane Database of Systematic Reviews	Singing for adults with chronic obstructive pulmonary disease (COPD)
Sereda et al.	2018	Cochrane Database of Systematic Reviews	Sound therapy (using amplification devices and/or sound generators) for tinnitus
Sinha et al.	2011	Cochrane Database of Systematic Reviews	Auditory integration training and other sound therapies for autism spectrum disorders (ASD)
van der Steen et al.	2018	Cochrane Database of Systematic Reviews	Music-based therapeutic interventions for people with dementia
Galaal et al.	2011	Cochrane Database of Systematic Reviews	Interventions for reducing anxiety in women undergoing colposcopy
Halsbeck et al.	2019	Cochrane Database of Systematic Reviews	Musical and vocal interventions to improve neurodevelopmental outcomes for preterm infants
Aalbers et al.	2017	Cochrane Database of Systematic Reviews	Music therapy for depression
Ghetti et al.	2020	Cochrane Database of Systematic Reviews	Music therapy for people with substance use disorders
Irons et al.	2019	Cochrane Database of Systematic Reviews	Singing for people with Parkinson's disease
Irons, Kenny & Chang	2010	Cochrane Database of Systematic Reviews	Singing for children and adults with bronchiectasis

© 2022 McCrary JM et al. *JAMA Network Open.*

Irons et al.	2019	Cochrane Database of Systematic Reviews	Singing as an adjunct therapy for children and adults with cystic fibrosis
Leckey	2011	Journal of Psychiatric and Mental Health Nursing	The therapeutic effectiveness of creative activities on mental well-being: A systematic review of the literature
Lee et a.	2015	Chest	Distractive Auditory Stimuli in the Form of Music in Individuals With COPD A Systematic Review
Lin et al.	2019	Journal of Clinical Medicine	Music interventions for anxiety in pregnant women: A systematic review and meta-analysis of randomized controlled trials
Magee et al.	2017	Cochrane Database of Systematic Reviews	Music interventions for acquired brain injury
Phillip, Lewis & Hopkinson	2019	Breathe	Music and dance in chronic lung disease

© 2022 McCrary JM et al. *JAMA Network Open.*

eTable 3. Details of GRADE ratings of included studies.

			GRADE Criteria							
**Author**	**Year**	**Study Design**	*Intention to treat analysis*	*All outcome results reported*	*Blinding (assessors/data analysts)*	*Allocation concealment*	*% lost to follow-up**	*Use of individual randomization*	*Controlled crossover effects*	**Overall quality rating**
Altena et al.[33]	2009	RCT	Yes	Yes	Yes	Yes	None	Yes	N/A	High
Ashok, Shanmugam & Soman[29]	2019	RCT	Yes	Yes	Unclear	Yes	Moderate (25%)	Yes	N/A	Moderate
Atiwannapat et al.[37]	2016	RCT	Yes	Yes	Yes	Unclear	Moderate (20%)	Yes	N/A	Moderate
Bittman et al.[43]	2020	RCT	No (*analysis only on those who attended at least 12 sessions*)	Yes	No	Unclear	Unclear	Yes	N/A	Low
Burrai et al.[30]	2020	RCT	Yes	Yes	Yes	Unclear	Low (10%)	Yes	N/A	Moderate
Corvo, Skingley & Clift[31]	2020	Single group study	Yes	Yes	No	No	Low (9%)	N/A	N/A	Low
Coulton et al.[39]	2015	RCT	Yes	Yes	Unclear	Yes	Moderate (20%)	Yes	N/A	Moderate
Davidson et al.[23]	2014	Single group study	Yes	Yes	N/A	N/A	Moderate (19%)	N/A	N/A	Very low
Gale et al.[40]	2012	Single group study	No (*those who missed 4+ rehearsals excluded*)	Yes	N/A	N/A	High (33%)	N/A	N/A	Very low
Groener et al.[28]	2015	RCT	Yes	Yes	Unclear	Unclear	None	Yes	N/A	Moderate
Hagemann, Martin & Neme[24]	2020	Single group study	Yes	Yes	N/A	N/A	Moderate (26%)	N/A	N/A	Very low
Innes et al.[44]	2018	RCT	Yes	Yes	Yes	Yes	None	Yes	N/A	High
Innes et al.[45]	2016	RCT	Yes	Yes	Yes	Yes	None	Yes	N/A	High
Lavretsky et al.[46]	2013	RCT	Yes	No (*only MCS and selected subscales of SF-36 reported*)	Yes	Yes	Moderate (20%)	Yes	N/A	Moderate
Lee, Chan & Mok[27]	2010	RCT	Yes	Yes	No	Yes	Low (11%)	Yes	N/A	Moderate
Logtenberg et al.[34]	2007	RCT	Yes	Yes	Yes	Yes	None	Yes	N/A	High

© 2022 McCrary JM et al. *JAMA Network Open.*

Lord et al.[45]	2010	RCT	Yes	Yes	Yes	Yes	Moderate (19%)	No (*block randomization*)	N/A	Moderate
Lord et al.[41]	2012	RCT	Yes	Yes	Yes	Yes	Moderate (28%)	No (*randomized in blocks of 4*)	N/A	Moderate
Mandel et al.[47]	2007	RCT	Yes	Yes	Unclear	Unclear	High (36%)	Yes	N/A	Low
Mateu et al.[35]	2018	Single group study	Yes	Yes	Unclear	Unclear	None	Yes	Yes	Moderate
Mujdeci et al.[38]	2015	Single group study	Yes	Yes	N/A	N/A	None	N/A	N/A	Low
Philip et al.[42]	2020	RCT	Yes	Yes	Yes	Yes	None	Yes	N/A	High
Ribeiro[25]	2018	RCT	No (*analysis only of those with >75% compliance*)	Yes	Unclear	Unclear	High	Yes	N/A	Low
Wahlstöm et al.[36]	2020	RCT	Yes	Yes	Unclear	Yes	High (34%)	No (*randomized in blocks of 6*)	N/A	Low
Zanini et al.[26]	2009	RCT	Yes	Yes	Unclear	Unclear	None	Unclear	N/A	Low
Zeppegno et al.[34]	2021	RCT	No (*4 of 30 patients excluded for 'low compliance'*)	Yes	Yes	Yes	None	Yes	N/A	Moderate

© 2022 McCrary JM et al. *JAMA Network Open.*

eFigure 1. PRISMA flow diagram detailing the results of record screening and exclusion procedures.[16]

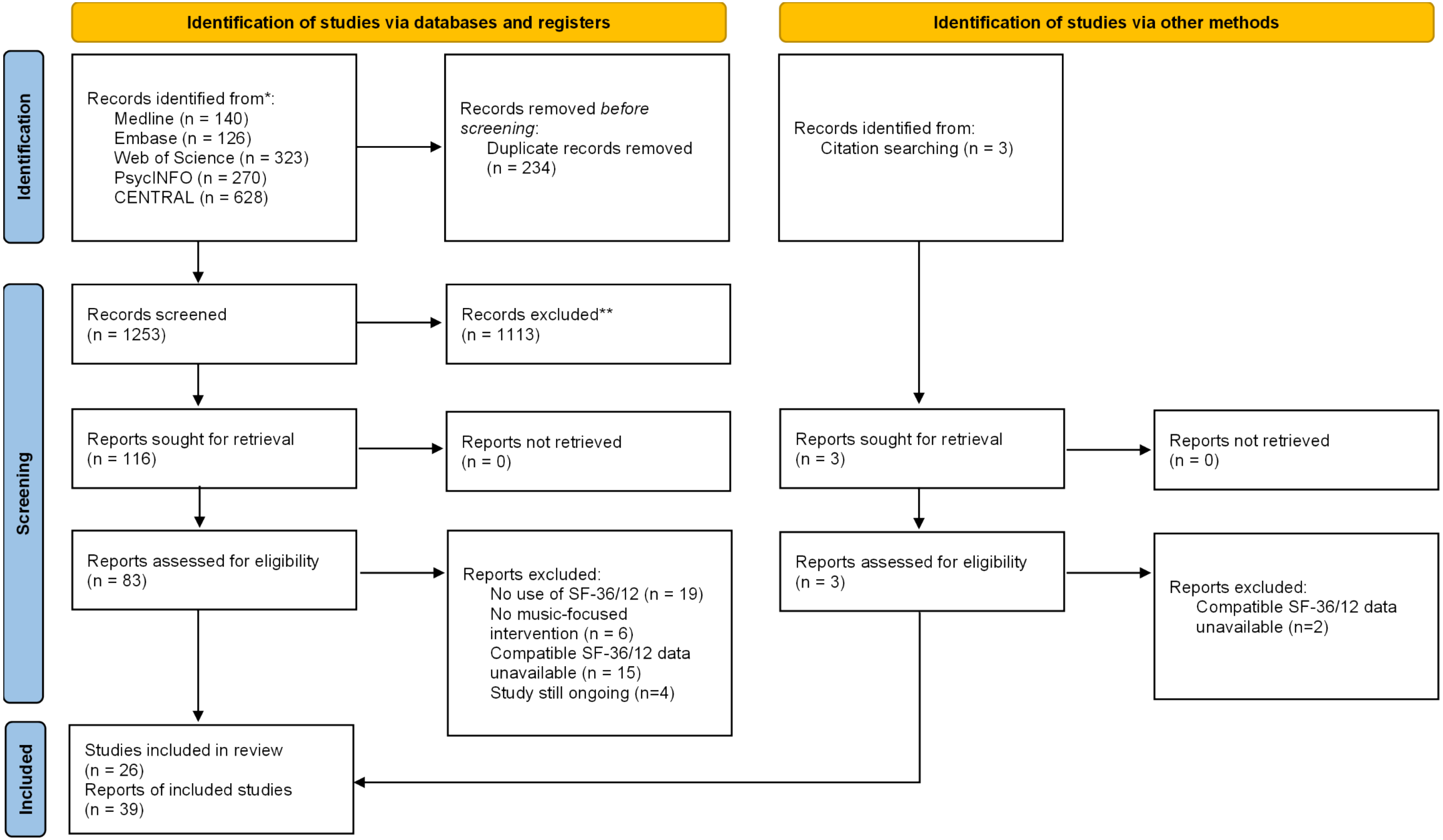

© 2022 McCrary JM et al. *JAMA Network Open.*

eFigure 2. Funnel plot detailing the distribution of pre-post intervention changes in MCS score, stratified by music intervention type. SE(MD) = Standard error of the mean difference. MD = Mean difference.

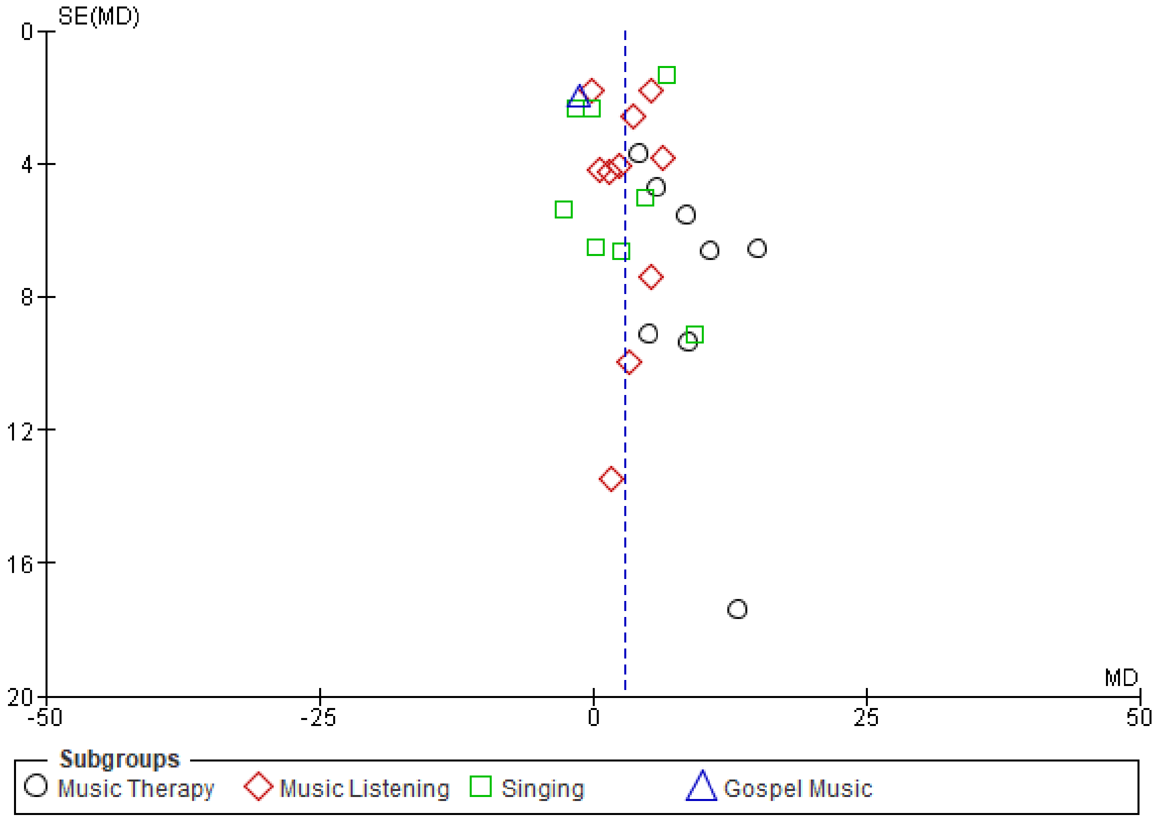

© 2022 McCrary JM et al. *JAMA Network Open.*

eFigure 3. Funnel plot detailing the distribution of pre-post intervention changes in PCS score, stratified by music intervention type. SE(MD) = Standard error of the mean difference. MD = Mean difference.

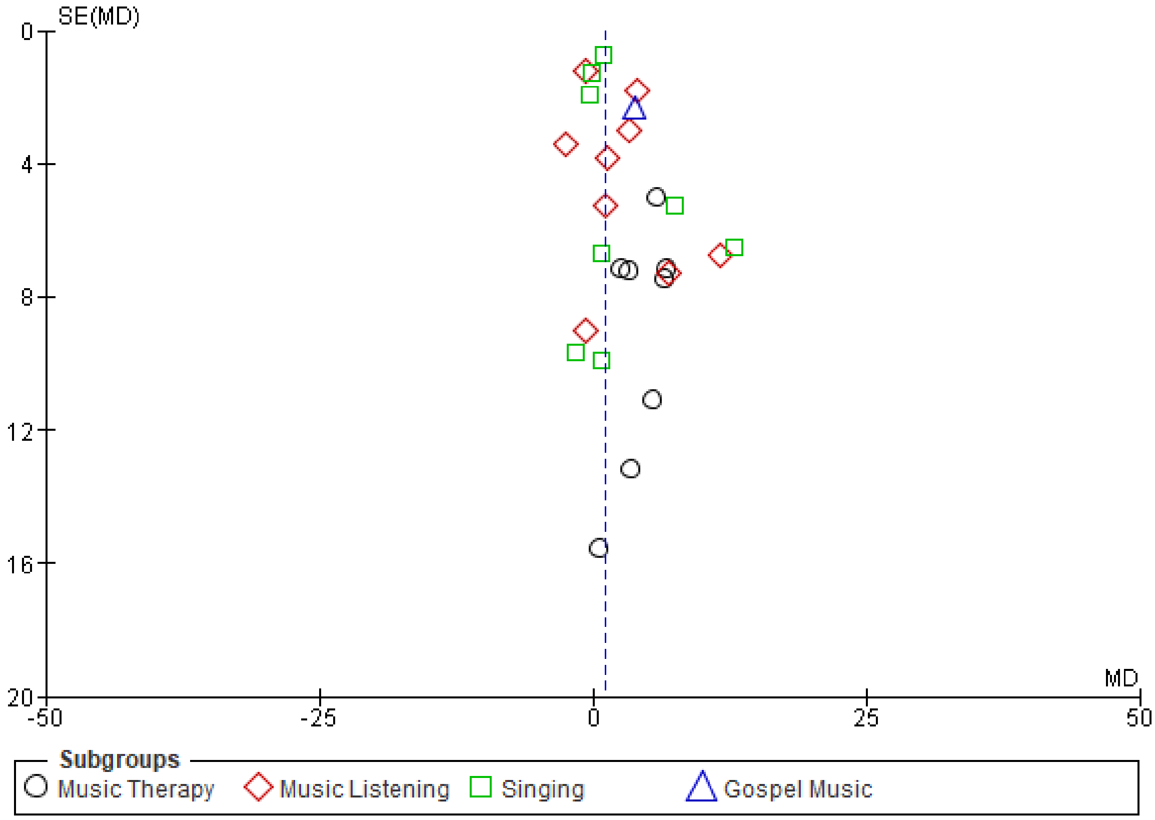

© 2022 McCrary JM et al. *JAMA Network Open.*

eFigure 4. Funnel plot detailing the distribution of changes in MCS scores in Music+TAU vs. TAU alone interventions, stratified by music intervention type. SE(MD) = Standard error of the mean difference. MD = Mean difference.

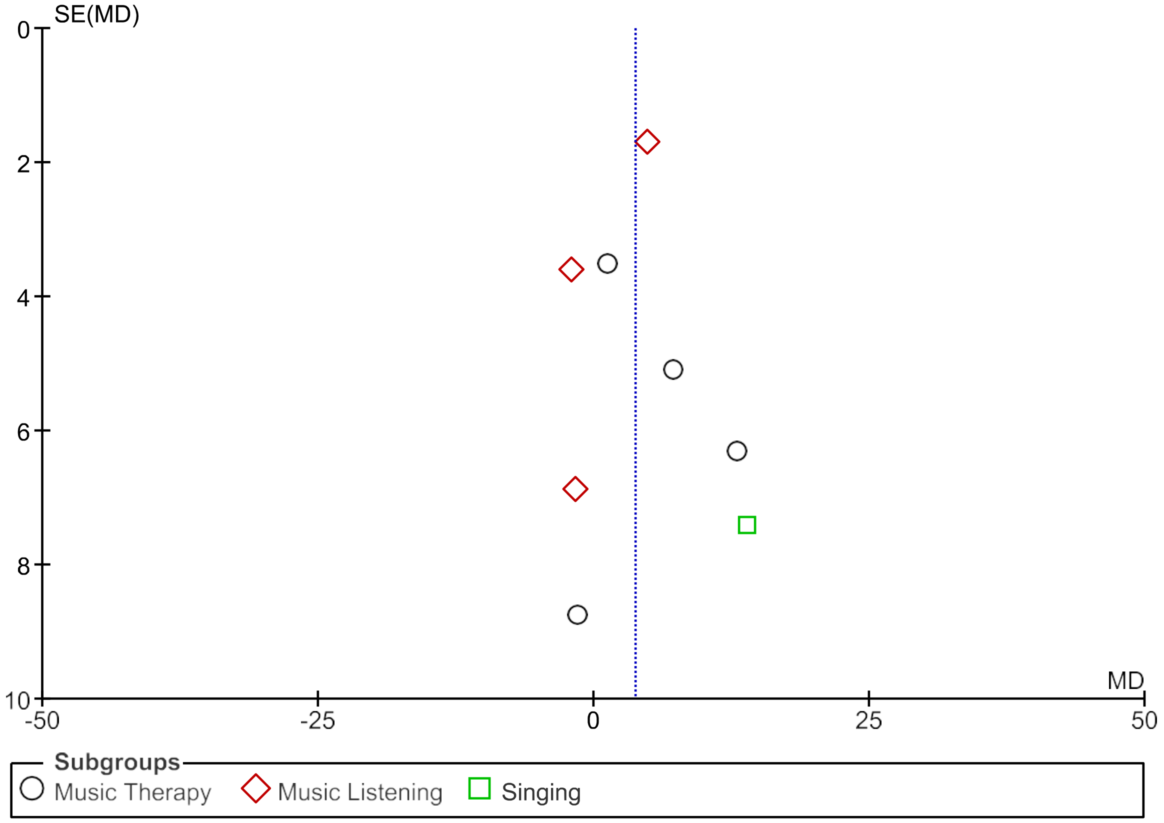

© 2022 McCrary JM et al. *JAMA Network Open.*

eFigure 5. Funnel plot detailing the distribution of changes in PCS scores in Music+TAU vs. TAU alone interventions, stratified by music intervention type. SE(MD) = Standard error of the mean difference. MD = Mean difference.

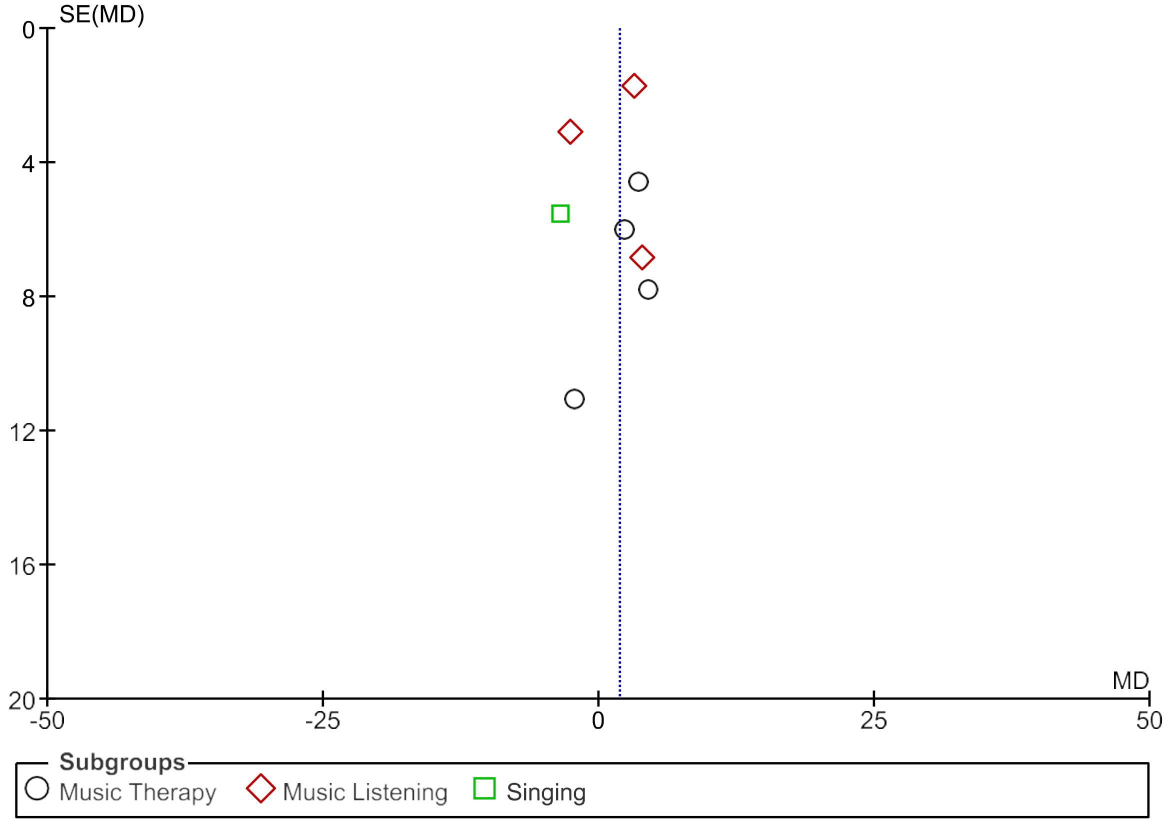

© 2022 McCrary JM et al. *JAMA Network Open.*

eFigure 6. Associations between music interventions and pre- to post-intervention changes in SF-36/SF-12 MCS scores, stratified by Music + TAU vs. all other music interventions. IV = 'inverse variance'. 'Total' refers to the total number of participants included in analyses at pre- and post-intervention timepoints.

	Post-intervention			Pre-intervention				Mean Difference
**Study or Subgroup**	**Mean**	**SD**	**Total**	**Mean**	**SD**	**Total**	**Weight**	**IV, Random, 95% CI**
**2.4.1 Music + TAU Interventions**								
Ashok, Shanmugam & Soman 2019	43.7	23.2	20	42.1	55.4	20	0.3%	1.60 [-24.72, 27.92]
Burrai et al. 2020	51.8	11.8	74	46.5	10.9	82	10.2%	5.30 [1.72, 8.88]
Mandel et al. 2007	52.2	20.1	55	48.1	18.8	55	3.8%	4.10 [-3.17, 11.37]
Phillip et al. 2020	53.8	15.6	9	53.6	11.7	9	1.4%	0.20 [-12.54, 12.94]
Ribeiro 2018	43	16	10	38	23.9	10	0.7%	5.00 [-12.83, 22.83]
Wahlstöm et al. 2020	44.2	16	29	43.6	16	29	3.1%	0.60 [-7.64, 8.84]
Zanini et al. 2009	57.8	11.3	23	49.3	24.1	23	1.9%	8.50 [-2.38, 19.38]
Zeppegno et al. 2021	50.8	16.1	26	45.1	17.8	26	2.5%	5.70 [-3.53, 14.93]
**Subtotal (95% CI)**			**246**			**254**	**23.9%**	**4.61 [1.97, 7.26]**
Heterogeneity: Tau² = 0.00; Chi² = 2.13, df = 7 (P = 0.95); I² = 0%								
Test for overall effect: Z = 3.42 (P = 0.0006)								
**2.4.2 All Other Music Interventions**								
Altena et al. 2009	78.8	36.4	15	75.6	12.8	15	0.6%	3.20 [-16.33, 22.73]
Atiwannapat et al. 2016 (Active MT)	35.6	8.67	4	26.9	18.5	5	0.7%	8.70 [-9.61, 27.01]
Atiwannapat et al. 2016 (Receptive MT)	46	26.8	4	32.8	24.6	5	0.2%	13.20 [-20.78, 47.18]
Bittman et al. 2020	52.8	9.9	36	54.1	6.8	36	9.2%	-1.30 [-5.22, 2.62]
Corvo, Skingley & Clift 2020	50	8.5	41	50.2	12.4	41	7.5%	-0.20 [-4.80, 4.40]
Coulton et al. 2015	55.5	9.3	105	48.8	11.5	131	13.4%	6.70 [4.05, 9.35]
Davidson et al. 2014	53.5	17.8	29	56.2	22.9	29	2.0%	-2.70 [-13.26, 7.86]
Gale et al. 2012	50.9	14	20	46.2	17.5	20	2.3%	4.70 [-5.12, 14.52]
Groener et al. 2015	50.5	6.3	18	52.1	7.7	18	7.5%	-1.60 [-6.20, 3.00]
Hagemann, Martin & Neme 2020	54.2	20.1	23	43.6	24.5	23	1.4%	10.60 [-2.35, 23.55]
Innes et al. 2016	75.3	12	11	70	21.3	11	1.1%	5.30 [-9.15, 19.75]
Innes et al. 2018	51.1	15.6	30	48.7	15.9	30	3.3%	2.40 [-5.57, 10.37]
Lavretsky et al. 2013	43.6	10.6	16	37.3	11	16	3.6%	6.30 [-1.19, 13.79]
Lee, Chan & Mok 2020	45.8	8.9	31	42.2	11.2	31	6.7%	3.60 [-1.44, 8.64]
Logtenberg et al. 2007	48.1	10.6	15	46.7	12.5	15	3.0%	1.40 [-6.89, 9.69]
Lord et al. 2010	49.9	20.9	15	47.4	14.8	15	1.4%	2.50 [-10.46, 15.46]
Lord et al. 2012	57.5	25.3	13	48.2	20.8	13	0.7%	9.30 [-8.50, 27.10]
Mateu et al. 2018	35.9	9.6	58	36.1	10.1	58	10.1%	-0.20 [-3.79, 3.39]
Mujdeci et al. 2015	45.1	12	13	30.1	20.3	13	1.4%	15.00 [2.18, 27.82]
**Subtotal (95% CI)**			**497**			**525**	**76.1%**	**2.59 [0.50, 4.69]**
Heterogeneity: Tau² = 6.30; Chi² = 28.79, df = 18 (P = 0.05); I² = 37%								
Test for overall effect: Z = 2.42 (P = 0.02)								
**Total (95% CI)**			**743**			**779**	**100.0%**	**2.95 [1.39, 4.51]**
Heterogeneity: Tau² = 2.89; Chi² = 32.58, df = 26 (P = 0.17); I² = 20%								
Test for overall effect: Z = 3.71 (P = 0.0002)								
Test for subgroup differences: Chi² = 1.38, df = 1 (P = 0.24), I² = 27.6%								

Mean Difference
IV, Random, 95% CI

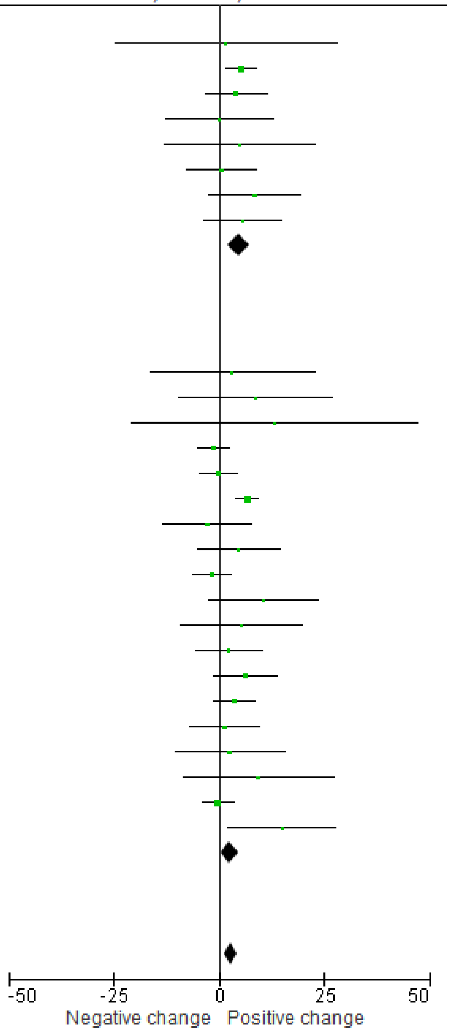

© 2022 McCrary JM et al. *JAMA Network Open.*

eFigure 7. Associations between music interventions and pre- to post-intervention changes in SF-36/SF-12 MCS scores, stratified by Music + TAU vs. all other music interventions. IV = 'inverse variance'. 'Total' refers to the total number of participants included in analyses at pre- and post-intervention timepoints.

	Post-intervention			Pre-intervention				Mean Difference
Study or Subgroup	Mean	SD	Total	Mean	SD	Total	Weight	IV, Random, 95% CI
**2.3.1 Music + TAU Interventions**								
Ashok, Shanmugam & Soman 2019	43.7	22.6	20	36.8	23.5	20	0.4%	6.90 [-7.39, 21.19]
Burrai et al. 2020	43.8	10.9	74	39.8	11.8	82	7.0%	4.00 [0.44, 7.56]
Mandel et al. 2007	44	25.7	55	38.3	27.1	55	0.9%	5.70 [-4.17, 15.57]
Phillip et al. 2020	30.8	11.8	9	32.4	26.4	9	0.2%	-1.60 [-20.49, 17.29]
Ribeiro 2018	46	23.2	10	40.5	26.1	10	0.2%	5.50 [-16.14, 27.14]
Wahlstöm et al. 2020	46.2	12	29	48.8	14	29	2.0%	-2.60 [-9.31, 4.11]
Zanini et al. 2009	46.8	19.5	23	40.1	28.1	23	0.5%	6.70 [-7.28, 20.68]
Zeppegno et al. 2021	48.8	20.5	26	45.6	30.6	26	0.4%	3.20 [-10.96, 17.36]
**Subtotal (95% CI)**			**246**			**254**	**11.6%**	**3.10 [0.34, 5.86]**
Heterogeneity: Tau² = 0.00; Chi² = 4.09, df = 7 (P = 0.77); I² = 0%								
Test for overall effect: Z = 2.20 (P = 0.03)								
**2.3.2 All Other Music Interventions**								
Altena et al. 2009	71.1	32	15	71.8	13.7	15	0.3%	-0.70 [-18.32, 16.92]
Atiwannapat et al. 2016 (Active MT)	43.6	17	4	43	29	5	0.1%	0.60 [-29.79, 30.99]
Atiwannapat et al. 2016 (Receptive MT)	47.6	19.3	4	44.2	19.9	5	0.1%	3.40 [-22.33, 29.13]
Bittman et al. 2020	47.9	10.1	36	44.1	9.8	36	4.2%	3.80 [-0.80, 8.40]
Corvo, Skingley & Clift 2020	38.9	6.1	41	39	5.6	41	13.7%	-0.10 [-2.63, 2.43]
Coulton et al. 2015	40	5	105	39.1	6.6	131	40.3%	0.90 [-0.58, 2.38]
Davidson et al. 2014	42	21	29	41.2	29.3	29	0.5%	0.80 [-12.32, 13.92]
Gale et al. 2012	43.2	29.8	20	42.4	32.7	20	0.2%	0.80 [-18.59, 20.19]
Groener et al. 2015	50.4	4.8	18	50.8	6.6	18	6.2%	-0.40 [-4.17, 3.37]
Hagemann, Martin & Neme 2020	45.7	23.6	23	43.1	24.8	23	0.5%	2.60 [-11.39, 16.59]
Innes et al. 2016	59.5	12.5	11	47.9	18.6	11	0.5%	11.60 [-1.64, 24.84]
Innes et al. 2018	46.5	16.6	30	45.5	23.6	30	0.8%	1.00 [-9.32, 11.32]
Lee, Chan & Mok 2020	35.8	14	31	34.6	15.9	31	1.6%	1.20 [-6.26, 8.66]
Logtenberg et al. 2007	43	9.5	15	39.7	6.6	15	2.6%	3.30 [-2.55, 9.15]
Lord et al. 2010	39.5	14.6	15	32	14	15	0.8%	7.50 [-2.74, 17.74]
Lord et al. 2012	49.4	19	13	36.5	13.8	13	0.5%	12.90 [0.13, 25.67]
Mateu et al. 2018	30	6.5	58	30.7	6.8	58	15.1%	-0.70 [-3.12, 1.72]
Mujdeci et al. 2015	55.4	16.1	13	48.9	21.5	13	0.4%	6.50 [-8.10, 21.10]
**Subtotal (95% CI)**			**481**			**509**	**88.4%**	**0.82 [-0.18, 1.82]**
Heterogeneity: Tau² = 0.00; Chi² = 13.08, df = 17 (P = 0.73); I² = 0%								
Test for overall effect: Z = 1.62 (P = 0.11)								
**Total (95% CI)**			**727**			**763**	**100.0%**	**1.09 [0.15, 2.03]**
Heterogeneity: Tau² = 0.00; Chi² = 19.49, df = 25 (P = 0.77); I² = 0%								
Test for overall effect: Z = 2.27 (P = 0.02)								
Test for subgroup differences: Chi² = 2.32, df = 1 (P = 0.13), I² = 56.9%								

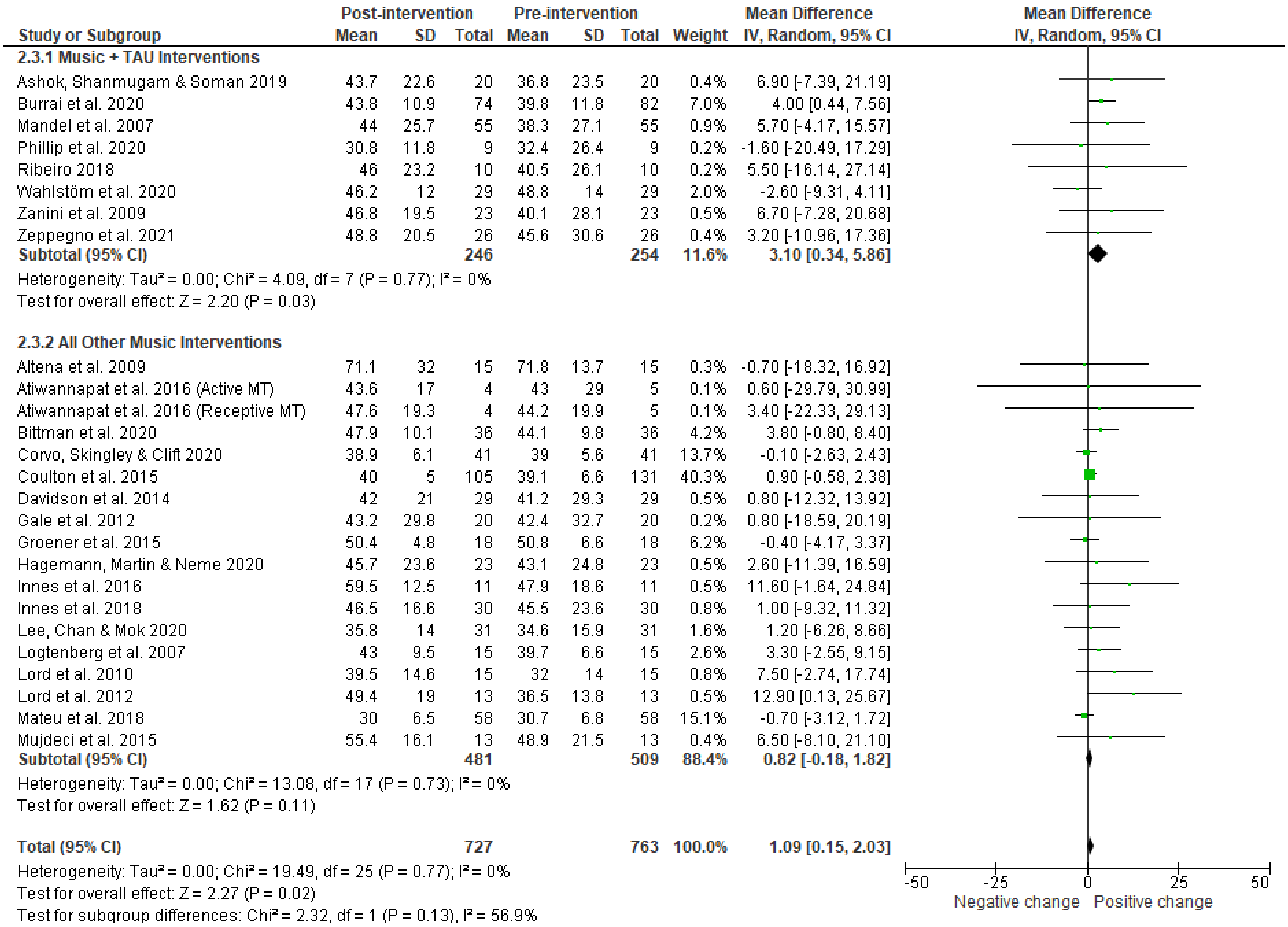

© 2022 McCrary JM et al. *JAMA Network Open.*

eFigure 8. Associations between music vs. meditation interventions and changes in SF-36/SF-12 MCS scores. IV = 'inverse variance'. 'Total' refers to the total number of participants included in analyses at pre- and post-intervention timepoints.

	Music Listening			Meditation				Mean Difference
**Study or Subgroup**	**Mean**	**SD**	**Total**	**Mean**	**SD**	**Total**	**Weight**	**IV, Random, 95% CI**
Innes et al. 2016	75.3	12	11	78.2	18.6	11	12.9%	-2.90 [-15.98, 10.18]
Innes et al. 2018	51.1	15.6	30	51.7	15.6	30	35.4%	-0.60 [-8.49, 7.29]
Lavretsky et al. 2013	43.6	10.6	16	47.2	9.7	23	51.7%	-3.60 [-10.13, 2.93]
**Total (95% CI)**			**57**			**64**	**100.0%**	**-2.45 [-7.15, 2.25]**

Heterogeneity: Tau² = 0.00; Chi² = 0.33, df = 2 (P = 0.85); I² = 0%
Test for overall effect: Z = 1.02 (P = 0.31)

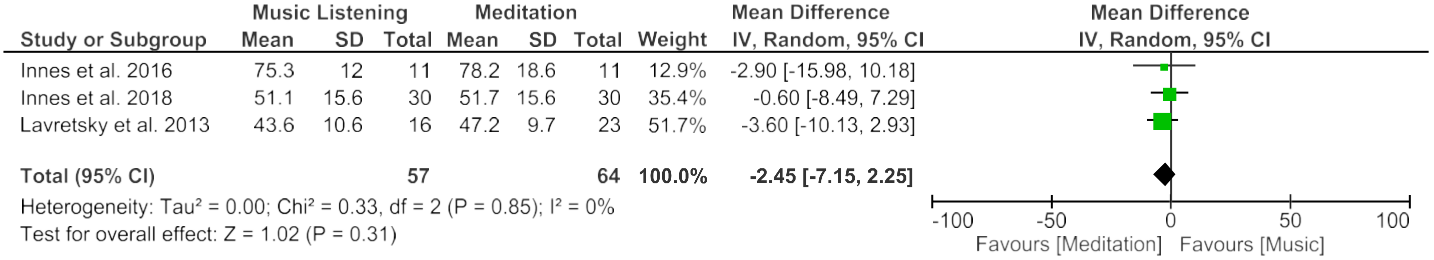

eFigure 9. Associations between music vs. meditation interventions and changes in SF-36/SF-12 PCS scores. IV = 'inverse variance'. 'Total' refers to the total number of participants included in analyses at pre- and post-intervention timepoints. NB: Lavretsky et al. 2013 reported MCS scores only.

	Music Listening			Meditation				Mean Difference
**Study or Subgroup**	**Mean**	**SD**	**Total**	**Mean**	**SD**	**Total**	**Weight**	**IV, Random, 95% CI**
Innes et al. 2016	59.5	12.5	11	66.4	17	11	34.9%	-6.90 [-19.37, 5.57]
Innes et al. 2018	46.5	16.6	30	45.9	19.4	30	65.1%	0.60 [-8.54, 9.74]
**Total (95% CI)**			**41**			**41**	**100.0%**	**-2.02 [-9.39, 5.35]**

Heterogeneity: Tau² = 0.00; Chi² = 0.90, df = 1 (P = 0.34); I² = 0%
Test for overall effect: Z = 0.54 (P = 0.59)

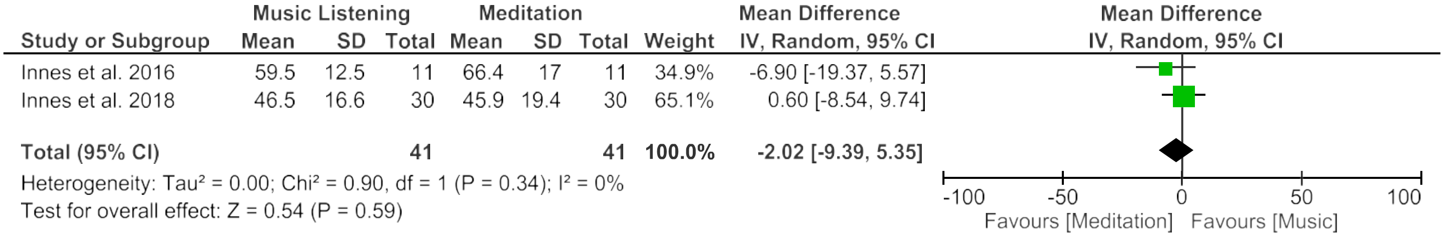

© 2022 McCrary JM et al. *JAMA Network Open.*
